# Drinking motives and alcohol sensitivity mediate multidimensional genetic influences on alcohol use behaviors

**DOI:** 10.1111/acer.70045

**Published:** 2025-03-31

**Authors:** Jeanne E. Savage, Spit for Science Working Group, Karen Chartier, Ananda Amstadter, Emily Lilley, Renolda Gelzinis, Anne Morris, Katie Bountress, Amy E. Adkins, Nathaniel Thomas, Zoe Neale, Kimberly Pedersen, Thomas Bannard, Seung B. Cho, Peter Barr, Holly Byers, Erin C. Berenz, Erin Caraway, Seung B. Cho, James S. Clifford, Megan Cooke, Elizabeth Do, Alexis C. Edwards, Neeru Goyal, Laura M. Hack, Lisa J. Halberstadt, Sage Hawn, Sally Kuo, Emily Lasko, Jennifer Lend, Mackenzie Lind, Elizabeth Long, Alexandra Martelli, Jacquelyn L. Meyers, Kerry Mitchell, Ashlee Moore, Arden Moscati, Aashir Nasim, Jill Opalesky, Cassie Overstreet, A. Christian Pais, Tarah Raldiris, Jessica Salvatore, Rebecca Smith, David Sosnowski, Jinni Su, Chloe Walker, Marcie Walsh, Teresa Willoughby, Madison Woodroof, Jia Yan, Cuie Sun, Brandon Wormley, Brien Riley, Fazil Aliev, Roseann Peterson, Bradley T. Webb, Danielle M. Dick, Danielle Posthuma

**Affiliations:** ^1^ Department of Complex Trait Genetics, Centre for Neurogenomics and Cognitive Research, Amsterdam Neuroscience Vrije Universiteit Amsterdam The Netherlands; ^2^ Department of Psychiatry, Robert Wood Johnson Medical School, Rutgers Addiction Research Center Rutgers University Piscataway New Jersey USA; ^3^ Department of Child and Adolescent Psychology and Psychiatry, Section Complex Trait Genetics, Amsterdam Neuroscience Vrije Universiteit Medical Center Amsterdam The Netherlands

**Keywords:** drinking motives, genetic heterogeneity, level of response to alcohol, mediation, polygenic scores

## Abstract

**Background:**

Genetic influences account for a substantial proportion of individual differences in alcohol use behaviors (AUBs). However, multiple distinct sets of genes are linked to different AUBs via uncertain causal links. Here, we explore whether intermediate neurobiological traits mediate the relationship between polygenic scores (PGSs) and multiple AUBs, with the aim to better understand processes captured by different genetic profiles.

**Methods:**

We derived four alcohol‐related PGSs in participants from Spit for Science, a longitudinal study of college students in the United States (*n* = 4549). Using linear regression, we tested the relationship between PGSs and 22 potential mediators, including personality, alcohol expectancies, drinking motives, and alcohol sensitivity. Nominally significant effects were carried forward to a multiple mediation model to estimate direct and indirect effects on four measured AUBs (frequency, quantity, alcohol use disorder symptoms [AUDsx], and maximum drinks in 24 h).

**Results:**

In univariable regression, PGSs indexing genetic effects on drinks per week (DPW) and problematic alcohol use (PAU) predicted higher levels of impulsivity and drinking motives as well as lower alcohol sensitivity. *BeerPref* PGSs (indexing a variable pattern of alcohol problems and preference for beer) predicted higher negative urgency and lower alcohol sensitivity. Mediational models indicated direct and indirect effects of DPW PGSs on multiple AUBs via social/enhancement drinking motives and alcohol sensitivity, indirect effects of PAU PGSs on AUDsx, and indirect effects of *BeerPref* PGS on drinking frequency and AUDsx via the joint effect of mediators including alcohol sensitivity.

**Conclusions:**

These findings provide evidence that the genetic influences on AUBs are associated with and partially mediated by intermediate neurobiological and cognitive factors, which may be more amenable to intervention. Greater focus on drinking motives and alcohol sensitivity is warranted in genetic research, as well as attention to the heterogeneous pathways linking genes to alcohol use outcomes.

## Graphical abstract

Alcohol use behaviors (AUBs) are heritable, and research has identified many regions of the genome that correlate with individual differences in AUBs. However, there are many steps between DNA and behavior where these genes could have an effect. Here, we tested whether genes are associated with AUBs via intermediate measures of personality, alcohol expectancies, drinking motives, and alcohol sensitivity. These intermediate pathways accounted for a substantial proportion of genetic effects, helping to clarify our understanding of how genes shape AUBs.

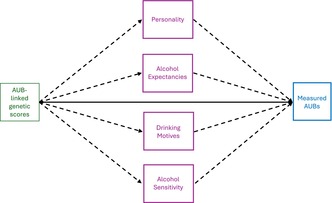

## INTRODUCTION

Alcohol use behaviors (AUBs) manifest in a variety of ways, with substantial individual and population differences in the frequency, quantity, timing, and types of alcohol that people consume—if they choose to drink at all (Litten et al., 2015; Rehm et al., 2003). AUBs also vary substantially across environments and throughout the lifespan. For example, individuals in Mediterranean countries typically consume moderate amounts of alcohol (primarily wine) and drink frequently but almost exclusively with meals, while individuals in northern and eastern European countries drink to intoxication more often and tend to drink beer and spirits (Sieri et al., 2002; Simpura & Karlsson, 2001). Young adults typically drink on fewer days but engage in higher levels of heavy episodic and risky drinking behaviors, while older adults consume smaller quantities of alcohol more frequently (Britton et al., 2015; Holton et al., 2019; Leggat et al., 2022). Clinically significant alcohol use disorders (AUDs) are marked by a dynamic progression toward heavier, uncontrolled drinking, although the tempo and intensity of this trajectory also differ between individuals (Prince & Maisto, 2013).

The factors influencing AUBs are as varied as the behaviors themselves. At the most basic level, genes have a robust impact on AUBs, with heritability estimates of 50%–60% for drinking frequency, quantity, maximum drinks in 24 h, alcohol problems, and lifetime AUD diagnoses (Agrawal et al., 2012; Dick et al., 2011; Verhulst et al., 2015). The effect sizes of individual single nucleotide polymorphisms (SNPs) in the DNA are generally extremely small and represent simple statistical associations showing that a certain allele is more common in individuals with a higher or lower level of AUBs (Deak & Johnson, 2021). There is no straightforward way of knowing whether this is due to a direct effect of a gene on AUBs, an indirect genetic effect on a trait/behavior that is genetically correlated with AUBs, or an indirect effect via some intermediary process.

Intermediate neurobiological traits such as personality, brain structure, and reward sensitivity correlate modestly, but consistently, with AUBs (Gunn et al., 2013; Whelan et al., 2014). More proximally, alcohol‐related cognitions, such as expectancies about the positive and negative effects of alcohol and motivations for drinking, as well as biological processes that shape alcohol metabolism and subjective feelings of intoxication (alcohol sensitivity) have robust and direct associations with AUBs (Agrawal et al., 2008; Bresin & Mekawi, 2021; Schuckit, 2009; Schuckit et al., 1997). There is some evidence that the genetic influences on AUBs are partially mediated by these intermediate factors (Kendler et al., 2021; Li et al., 2017; Littlefield et al., 2011; Prescott et al., 2004). However, such mediational studies are few and generally apply biometrical analyses to infer genetic influences from twin correlations without providing molecular genetic insight. A few studies have linked specific candidate genes or aggregate genetic risk factors for related constructs (e.g., attention deficit hyperactivity disorder [ADHD] and risky behavior) to AUBs via measures of brain structure or connectivity (Aydogan et al., 2021; Carey et al., 2017; Hagerty et al., 2020; Villafuerte et al., 2013). However, there are now hundreds of genomic risk loci linked to AUBs (Saunders et al., 2022; Zhou et al., 2023), for which the mechanism of association remains largely unexplained.

Studies that seek to identify AUB‐related genes or link these to intermediate neurobiology generally also focus on only a few alcohol measures, lumping these into broad dimensions such as overall (current) consumption levels or lifetime AUD diagnoses. Emerging evidence from genome‐wide association studies (GWASs), however, has demonstrated that the relationships among AUBs are complex. While “alcohol consumption” and “alcohol problems” form consistent, coherent dimensions, they are genetically distinct from each other (Deak & Johnson, 2021; Sanchez‐Roige et al., 2019). A recent study using a multidimensional approach identified four distinct latent genetic factors underlying a set of 18 normative and problematic AUBs in the UK Biobank sample (Savage et al., 2024). Two of these, *Consumption* and *Problems*, were highly genetically correlated with measures investigated in prior GWASs of typical drinks per week (DPW) and problematic alcohol use (PAU), respectively. Two other factors were more complex, capturing novel genetic influences not seen in studies of unidimensional AUBs. The factor called *BeerPref* included high genetic loadings on measures such as quantity of beer (but not wine) consumption, drinking without accompanying meals, clinical consequences such as alcohol‐related health conditions, receiving advice from a medical professional to cut down on drinking, and a self‐reported decrease in drinking over the past 10 years (in a sample of middle‐aged and older adults). The *AtypicalPref* factor was indexed by high loadings on quantity of consumption of uncommon types of beverages such as fortified wine and spirits. These four factors were genetically distinct from each other (*r*_g_ = −0.33 to 0.56) and showed different patterns of associations with a variety of traits, behaviors, brain regions, and cell types (Savage et al., 2024).

The heterogeneity of AUBs and their underlying causes poses a challenge for efforts to develop personally tailored prevention and intervention efforts (Litten et al., 2015). Personalized strategies can be more effective than universal programs for reducing risky or harmful AUBs (Cronce & Larimer, 2011; Edalati & Conrod, 2018; Savage et al., 2015; Schuckit et al., 2012). This is especially critical as prevention programs often have small effects on reducing alcohol use (Strøm et al., 2014), and relapse is a prominent feature of AUD treatment (Sliedrecht et al., 2019). Incorporating genetic predictions, in the form of polygenic scores (PGSs), is an ambition for many personalized medicine applications (Lewis & Vassos, 2020) as DNA has desirable qualities for an intervention target. One's genetic code is not subject to reverse causation, so the direction of effect is clear; DNA is fixed before birth, so it is possible to identify at‐risk individuals prior to the onset of problems; and one's genome provides the ultimate level of individual‐specific prediction (save for monozygotic twins). Yet, genetic instruments are not commonly used in personalized interventions for AUBs, in no small part due to the fact that our current knowledge of the links between DNA variation and AUBs provides very poor individual‐level accuracy (<5%) in AUB prediction (Saunders et al., 2022; Zhou et al., 2023). However, this low accuracy may itself be a result of heterogeneity in the phenotype definition. As both gene discovery and PGS validation efforts are based almost exclusively on broad consumption and AUD phenotypes, genetic effects specific to different AUBs may be washed out, even as larger discovery sample sizes allow for the identification of more and more associated genes with smaller and smaller effects (Deak & Johnson, 2021).

One solution to this challenge is to focus gene identification efforts on more diverse and refined measures of AUBs (Mallard et al., 2022; Savage et al., 2024; Wong & Schumann, 2008), which provide better insight into their genetic architecture. It requires much effort and a high participant burden to collect such measures at scale, so it may take time to develop large datasets with deep phenotyping. In the meantime, however, PGSs can be applied not only for direct prediction of measured AUBs but also to investigate intermediate processes connecting genes to AUBs (Li et al., 2017; Salvatore et al., 2014). In combination with more refined gene discovery efforts, PGSs have the potential to identify more proximal processes, such as alcohol expectancies and drinking motives, through which genes exert their effects. Such constructs are much more amenable to manipulation than the DNA itself and have been demonstrated to be effective targets for intervention (Hingson, 2010). By linking genetic factors to precise intermediate processes, a greater level of specificity could also be achieved for developing individually tailored interventions—predicting not just whether someone is at higher or lower genetic risk but the actual process of *why* they might be at risk. Knowing which prevention or treatment is best suited to an individual could improve outcomes and reduce the amount of time spent in unsuccessful treatment attempts, along with negative health and societal consequences during those periods.

In this study, we investigate the intermediate mechanisms underlying genetic influences on multiple dimensions of AUBs. Applying a multiple mediation model, we examine the parallel role of drinking motives, alcohol expectancies, alcohol sensitivity, and personality traits linking individual differences in the genome to individual differences in AUBs. These findings have the potential to provide better insight into the statistical associations identified in GWAS as well as guiding future applications of interventions based on individual‐level genetic risk.

## MATERIALS AND METHODS

### Participants

Data were derived from “Spit for Science” (S4S), a prospective longitudinal study of over 12,000 college students at a large, urban, public university (Dick et al., 2014). Across multiple incoming student cohorts, all first‐time freshmen aged 18+ were eligible to complete a self‐report survey and provide a saliva sample for DNA collection early in the fall of their first year (Y1F). Follow‐up surveys were sent out each subsequent spring to participants still enrolled at the university (Y1S‐Y4S). Participants were 62.7% (cis gender) female and 48.6% self‐reported their race and ethnicity as White. All participants provided informed consent, and the S4S study was approved by the university Institutional Review Board.

After applying inclusion criteria (below), this study used data from *N* = 4549 S4S participants from the first five cohorts for whom genotyping was complete. Enrollment rates were high, with 64% of the eligible incoming students completing an initial survey and 42%–73% returning to complete follow‐up surveys across the subsequent data collection waves. Nearly all participants, 97%, provided a DNA sample. Participants in the current study were restricted to those whose genomes were most similar to European ancestry reference panels and whose DNA samples passed genetic quality control thresholds (Dick et al., 2014; Peterson et al., 2017; Webb et al., 2017). Ancestry filtering was applied due to the need to match ancestral background to the European discovery GWAS sample, as there is poor genetic prediction across ancestry groups due to inherited differences in patterns of linkage disequilibrium (LD) in the genome.

### Measures

Eligible participants were emailed a link to complete a confidential online self‐report survey that assessed a wide range of traits and behaviors, with a focus on alcohol use. Data were collected and managed by the secure, web‐based REDCap system of electronic data capture tools (Harris et al., 2009). Measures were largely derived from psychometrically validated scales administered in an abbreviated version or staggered across waves/cohorts to reduce participant burden. Psychometric properties and further details of most scales in this sample have been published previously (Dick et al., 2014; Salvatore et al., 2016; Savage et al., 2022; Savage & Dick, 2023a). Descriptive statistics for each of the measures are shown in Table 1. Stable traits such as personality were assessed in the initial surveys (Y1F and Y1S), while alcohol‐related measures were collected at every wave. For Cohort 5, only data through Y2S were used due to the onset of the COVID‐19 pandemic in Y3S and subsequent changes in both the campus environment and study protocols.

**TABLE 1:** Descriptive statistics of alcohol use behavior (AUB) measures and mediators.

	*N*	Mean	SD	Min	Max	Skew	Kurtosis
Personality							
Agreeableness	3821	12.01	2.15	3	15	−0.62	0.17
Conscientious	3822	13.17	1.87	4	15	−1.32	2.03
Extroversion	3822	10.68	2.98	3	15	−0.43	−0.61
Neuroticism	3821	8.60	2.98	3	15	0.06	−0.73
Openness	3822	12.82	2.05	3	15	−1.03	0.83
Lack of perseverance	4306	1.71	0.55	1	4	0.70	0.40
Lack of premeditation	4307	1.83	0.59	1	4	0.50	0.07
Negative urgency	4304	2.24	0.74	1	4	0.22	−0.66
Positive urgency	4301	2.01	0.71	1	4	0.49	−0.38
Sensation seeking	4306	2.95	0.69	1	4	−0.44	−0.32
Resilience	2974	6.13	1.48	0	8	−0.70	0.29
Expectancies							
Cognitive behavioral impairment	3595	3.12	0.70	1	4	−0.76	0.28
Enhanced sexuality	3455	2.11	1.00	1	4	0.42	−1.03
Liquid courage	3592	2.92	0.86	1	4	−0.57	−0.47
Self‐perception	3596	2.07	0.81	1	4	0.47	−0.61
Sociability	3588	3.51	0.71	1	4	−1.73	2.87
Tension reduction	3596	2.71	0.78	1	4	−0.32	−0.52
Motives							
Conformity	4005	1.49	0.64	1	4	1.47	1.71
Coping	3994	1.99	0.85	1	4	0.53	−0.69
Enhancement	3998	2.99	0.72	1	4	−0.87	0.61
Social	4003	3.06	0.71	1	4	−0.90	0.72
Alcohol sensitivity	3839	5.64	2.57	1	19	0.91	1.02
AUBs							
Drinking frequency	4239	3.91	3.59	0	16	1.26	0.98
Drinking quantity	4228	3.47	2.01	0	10	0.44	0.08
AUD symptoms	4236	2.02	2.05	0	11	1.12	0.80
Max drinks in 24 h	4080	10.96	6.04	1	25	0.59	−0.18

#### Personality traits

The surveys assessed personality traits using a subset of items for each subscale of the Big Five Inventory (BFI; John & Srivastava, 1999), each impulsivity‐related subscale (sensation seeking, negative urgency, positive urgency, lack of premeditation, and lack of perseverance) of the UPPS‐P (Lynam et al., 2006), and the Connor–Davidson resilience scale (CD‐RISC; Connor & Davidson, 2003). Prorated sum scores were available for the BFI subscales in Cohorts 1–4. Mean scores were available for the UPPS‐P subscales for all cohorts and for the CD‐RISC scale in Cohorts 1–3.

#### Alcohol expectancies

In the freshman and sophomore year waves (Y1F‐Y2S), participants were asked about what effects they expected to experience from drinking alcohol (whether or not they had yet initiated use). These included six subscales of Cognitive Behavioral Impairment, Enhanced Sexuality, Liquid Courage, Sociability, Tension Reduction, and Self‐Perception from the Alcohol Expectancies Questionnaire (AEQ; Fromme et al., 1993). Subscale scores were averaged across waves to create a single mean score.

#### Drinking motives

In each survey, participants who had initiated drinking (>1 full drink in their lifetime) completed an abbreviated version of the Drinking Motives Questionnaire—Revised (Cooper, 1994). Mean scores were derived for four subscales: Coping, Enhancement, Conformity, and Social motives. Subscale scores were averaged across waves.

#### Alcohol sensitivity

In each survey, participants who reported having used alcohol five or more times in their lives (85.8%) responded to the Self‐Rating of the Effects of Alcohol (SRE) scale (Schuckit et al., 1997). This scale consists of four questions that ask students to think back to the first five times they consumed alcohol and report how many standard drinks it took for them to feel tipsy/have a buzz, feel dizzy/slur their speech, stumble/find it hard to walk, and fall asleep without intending to. The average number of drinks needed to feel these intoxicating effects was used to define the SRE, with lower values reflecting a higher alcohol sensitivity. SRE scores from the first available self‐report were used for analysis to ensure that recall was as close in time as possible to the initiation of alcohol use.

#### Alcohol use behaviors

At each wave, participants who reported having used alcohol five or more times in their lives (85.8%) were asked about a variety of AUBs. These included questions about typical drinking frequency (number of drinking days per month; Freq) and typical drinking quantity (number of drinks per drinking day; Quant) from the AUDIT questionnaire (Bohn et al., 1995), *DSM‐5* AUD symptoms (AUDsx) from the Semi‐Structured Assessment for the Genetics of Alcoholism (SSAGA; Bucholz et al., 1994), and maximum drinks consumed in a 24‐h period in the past year (Max24), also from the SSAGA. Frequency and quantity measures were recoded to a pseudo‐continuous number of days per month or drinks per day using the mean of each range category or the most conservative value when the range was non‐specific (e.g., 16 days for the category “4 or more times a week”). Mean values of Freq, Quant, and AUDsx across waves, and maximum values of Max24, were used for analysis.

### Polygenic scores (PGSs)

Four PGSs were derived from summary statistics of previously published studies: the largest available GWASs of DPW (Saunders et al., 2022) and PAU (Zhou et al., 2023) and the *BeerPref* and *AtypicalPref* factors from a prior genomic structural equation model (Savage et al., 2024). As described above, these were selected because they represent genetically distinct factors underlying a wide variety of AUBs.

For each of these four measures, we created PGSs based on the GWAS summary statistics. PGS weights were created using PRS‐CS “auto” version and European LD reference panels from UK Biobank (*BeerPref; AtypicalPref*) or 1000 Genomes (DPW; AUD), as provided with the software (Ge et al., 2019). PGSs were calculated in PLINK2 (Chang et al., 2015) using the default (average) ‐‐score method. SNPs in the S4S dataset were first filtered on imputation INFO score >0.8, minor allele frequency >0.05, Hardy–Weinberg equilibrium *p* values >5 × 10^−6^, and missingness <0.025. Full details about genotyping and quality control procedures for this sample have been described elsewhere (Dick et al., 2014; Webb et al., 2017). Each PGS was entered in a regression model with 10 ancestry principal components, genetic sex, and age (mean across available data waves) as predictors. The standardized PGS residuals, after removing the effects of these covariates, were used for further analysis.

### Data analysis

**FIGURE 1:**
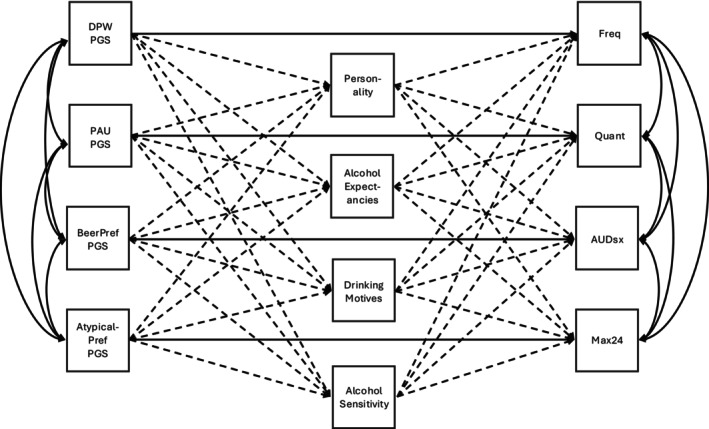
Example diagram of the multiple mediation model linking polygenic scores (PGSs) to alcohol use behaviors. Solid lines represent direct effects and dashed lines represent indirect effects. Single‐headed arrows represent directional association pathways and double‐headed arrows represent correlations.

All measures were standardized prior to analysis. First, we examined the overlap of genetic information between the four PGSs by calculating pairwise Pearson's correlations between each PGS. Second, we conducted a series of univariable linear regression analyses to identify which AUBs and potential mediators demonstrated evidence of association with each PGS. These analyses were carried out with the lm() function in R version 4.2.2 (R Core Team, 2017). As GWAS results give no indication about the underlying process responsible for the associations observed between genetic variants and AUBs, we used these exploratory analyses to guide the selection of plausible mediators. Nominally significant associations (*p* < 0.05) between each PGS and measures of personality, alcohol expectancies, drinking motives, or alcohol sensitivity were selected for the full model. Finally, these selected associations were included as mediational paths linking PGSs to each measured AUB in a multiple mediation model (Figure 1), while accounting for the correlations between these variables. Mediational analyses were carried out with structural equation modeling in Mplus version 8.4 (Muthén & Muthén, 2011), using robust maximum likelihood estimation to account for missing and nonnormally distributed data. The models provided estimates of the effect of each PGS on each AUB outcome, from both direct paths and indirect effects via the mediators. Bonferroni correction for four PGSs and four AUB outcome measures was applied (0.05/(4 × 4) = 0.003) to evaluate the significance of these direct and indirect effects in the mediation model.

## RESULTS

Correlations between the four PGSs ranged from 0.104 (DPW–BeerPref) to 0.526 (DPW–PAU), reflecting the heterogeneity of genetic influences captured by these different scores (Table 2). Univariable regression results demonstrated that most AUBs and potential mediators had a nominal association (*p* < 0.05) with at least one PGS, although the patterns were varied (Table 3). DPW and PAU were both highly significantly associated with higher levels of all AUBs and drinking motives (except conformity motives), as well as multiple impulsivity traits, alcohol sensitivity, and alcohol expectancies. A few dimension‐specific associations were also observed, such as between DPW and higher lack of perseverance, and between PAU and higher negative urgency. *BeerPref* predicted higher extroversion, lower expectations of cognitive behavioral impairment from alcohol, lower alcohol sensitivity, lower conformity motives, and higher Quant, AUDsx, and Max24. *AtypicalPref* was associated only with lower conformity motives and a lower alcohol sensitivity. Perhaps surprisingly, expectations of tension reduction effects from alcohol were not linked to any alcohol‐related PGS.

**TABLE 2:** Genetic correlations between polygenic scores for alcohol use behaviors.

	1	2	3	4
1. DPW	1.000			
2. PAU	0.526	1.000		
3. BeerPref	0.104	0.232	1.000	
4. AtypicalPref	0.242	0.117	0.259	1.000

Abbreviations: DPW, drinks per week; PAU, problematic alcohol use.

**TABLE 3:** Univariable regression associations between polygenic scores (PGSs) and alcohol use behavior (AUB) measures and mediators.

	Outcome	DPW		PAU		BeerPref		AtypicalPref	
		*β*	*p*	*β*	*p*	*β*	*p*	*β*	*p*
Personality	Agreeableness	**−0.039**	**0.016**	−0.029	0.073	−0.027	0.109	−0.021	0.191
	Conscientious	**−0.032**	**0.048**	−0.009	0.581	0.008	0.652	0.008	0.638
	Extroversion	0.016	0.310	0.014	0.373	**0.048**	**0.005**	0.008	0.631
	*Neuroticism*	*−0.021*	0.194	*0.017*	0.287	*0.033*	0.054	*−0.017*	0.298
	*Openness*	*0.021*	0.193	*0.023*	0.156	*0.008*	0.652	*0.025*	0.124
	Lack of perseverance	**0.053**	**4.6E‐04**	0.017	0.277	−0.027	0.076	−0.013	0.397
	Lack of premeditation	**0.030**	**0.047**	0.017	0.268	0.028	0.071	0.016	0.297
	Negative urgency	0.019	0.223	**0.047**	**0.002**	**0.047**	**0.002**	0.010	0.518
	Positive urgency	**0.051**	**7.9E‐04**	**0.051**	**9.3E‐04**	0.015	0.329	0.012	0.447
	Sensation seeking	**0.038**	**0.012**	**0.044**	**0.004**	0.010	0.510	0.019	0.219
	*Resilience*	*0.002*	*0.897*	*0.016*	*0.372*	*−0.020*	*0.304*	*−0.003*	*0.876*
Expectancies	Cognitive behavioral impairment	−0.030	0.072	−0.019	0.258	**−0.050**	**0.003**	−0.016	0.325
	Enhanced sexuality	0.033	0.051	**0.044**	**0.009**	0.023	0.174	0.012	0.471
	Liquid courage	**0.034**	**0.038**	**0.047**	**0.004**	0.013	0.456	−0.003	0.860
	*Self‐perception*	*0.003*	*0.838*	*0.007*	*0.664*	*−0.028*	*0.101*	*−0.002*	*0.910*
	Sociability	0.023	0.160	**0.037**	**0.026**	0.005	0.786	−0.005	0.742
	*Tension reduction*	*0.003*	*0.856*	*0.002*	*0.909*	*0.025*	*0.139*	*−0.003*	*0.857*
Motives	Conformity	0.010	0.545	0.021	0.177	**−0.036**	**0.022**	**−0.035**	**0.026**
	Coping	**0.058**	**2.7E‐04**	**0.065**	**4.0E‐05**	0.013	0.419	0.006	0.695
	Enhancement	**0.079**	**6.2E‐07**	**0.052**	**1.1E‐03**	0.014	0.107	−0.012	0.466
	Social	**0.085**	**8.4E‐08**	**0.056**	**4.1E‐04**	0.025	0.360	−0.012	0.462
Alcohol sensitivity		**0.047**	**0.004**	**0.037**	**0.022**	**0.046**	**0.005**	**0.044**	**0.006**
AUBs	Freq	**0.126**	**2.3E‐16**	**0.084**	**5.6E‐08**	0.024	0.118	0.013	0.392
	Quant	**0.081**	**1.4E‐07**	**0.043**	**0.006**	**0.033**	**0.032**	0.004	0.782
	AUDsx	**0.089**	**7.0E‐09**	**0.091**	**3.6E‐09**	**0.040**	**0.009**	0.007	0.637
	Max24	**0.084**	**8.0E‐08**	**0.055**	**5.0E‐04**	**0.039**	**0.014**	0.020	0.196

*Note*: Standardized effect sizes are presented. Italicized measures were not associated with any PGS and were not included in the full model. Bold values were nominally significant (*p* < 0.05).

Abbreviations: AUDsx, alcohol use disorder symptom count; DPW, drinks per week; Freq, drinking frequency; Max24, maximum drinks in 24 h; PAU, problematic alcohol use; Quant, drinking quantity.

Nominally associated variables from the univariable regressions were carried forward to a multiple mediation model. The full model showed that measured AUBs were also quite distinct from each other, with correlations ranging from 0.119 (Quant–AUDsx) to 0.258 (Freq–AUDsx). The full model explained between 14.3% and 35.7% of the variance in each of the measured AUBs (Table 4). However, this was mostly driven by the mediators themselves, as the direct and indirect effects of the PGSs were small (maximum standardized *β* = 0.071). DPW PGSs had significant direct effects (standardized *β* = 0.071) on Freq and indirect effects on all AUBs (standardized *β* = 0.034–0.056), which were specifically mediated through higher levels of positive reinforcement drinking motives (enhancement and social) and a lower level of alcohol sensitivity. PAU PGSs, on the other hand, only had significant total indirect effects on AUDsx. These effects could not be distinguished between specific mediators, although the largest effect size was observed for coping motives (standardized *β* = 0.010, *p* = 0.017). *BeerPref* PGSs had significant indirect effects (total standardized *β* = 0.014–0.019) on higher levels of Freq and AUDsx, although the specific indirect pathways were not statistically distinguishable from each other.

**TABLE 4:** Direct and indirect effects of polygenic scores (PGSs) for latent factors of alcohol use behavior (AUB) measures predicting AUBs in multiple mediation models.

PGS	Outcome	Freq	Quant	AUDsx	Max24
Total *R* ^2^		0.180	0.294	0.143	0.357
DPW	Total effects	0.117*	0.090*	0.063*	0.080*
	Direct	0.071*	0.034	0.029	0.012
	Total indirect	0.046*	0.056*	0.034*	0.054*
	Specific indirect				
	Agreeableness	0.000	0.001	0.000	0.002
	Conscientiousness	0.001	0.000	0.001	0.000
	Lack of perseverance	0.001	0.001	0.002	0.002
	Lack of premeditation	0.001	0.001	0.001	0.001
	Positive urgency	0.001	0.001	0.002	0.001
	Sensation seeking	0.002	0.002	0.000	0.003
	Liquid courage	0.000	0.001	0.001	0.001
	Coping motives	0.003	0.000	0.007	0.002
	Enhancement motives	0.014*	0.017*	0.007	0.011*
	Social motives	0.016*	0.018*	0.009*	0.013*
	Alcohol sensitivity	0.006	0.015	0.004	0.020*
PAU	Total effects	0.029	−0.005	0.059*	0.004
	Direct	0.012	−0.023	0.031	−0.016
	Total indirect	0.017	0.018	0.028*	0.020
	Specific indirect				
	Negative urgency	0.000	0.000	0.003	−0.001
	Positive urgency	0.001	0.001	0.002	0.001
	Sensation seeking	0.003	0.002	0.001	0.004
	Liquid courage	0.001	0.002	0.005	0.002
	Sexuality	0.000	0.001	0.002	0.000
	Sociability	0.000	0.001	0.001	0.001
	Coping motives	0.005	0.000	0.010	0.003
	Enhancement motives	0.003	0.003	0.001	0.002
	Social motives	0.003	0.003	0.002	0.002
	Alcohol sensitivity	0.002	0.004	0.001	0.005
BeerPref	Total effects	0.007	0.029	0.029	0.034
	Direct	−0.013	0.009	0.015	0.008
	Total indirect	0.019*	0.020	0.014*	0.025
	Specific indirect				
	Extroversion	0.005	0.002	0.004	0.002
	Negative urgency	0.000	0.000	0.003	−0.001
	Cognitive behavioral impairment	0.006	0.002	0.003	0.003
	Conformity motives	0.002	0.001	−0.001	0.001
	Alcohol sensitivity	0.006	0.015	0.004	0.019
AtypicalPref	Total effects	−0.007	−0.011	−0.002	0.006
	Direct	−0.014	−0.024	−0.014	−0.010
	Total indirect	0.007	0.012	0.002	0.016
	Specific indirect				
	Conformity motives	0.002	0.001	−0.001	0.001
	Alcohol sensitivity	0.005	0.011	0.003	0.015

*Note*: Standardized effects presented.

Abbreviations: AUDsx, alcohol use disorder symptom count; DPW, drinks per week; Freq, drinking frequency; Max24, maximum drinks in 24 h; PAU, problematic alcohol use; Quant, drinking quantity.

* *p* < 0.05/(4 PGS × 4 AUBs) = 0.003.

## DISCUSSION

Combining large‐scale, multidimensional gene discovery efforts with mediational modeling in a deeply phenotyped sample, this study has demonstrated that aggregated genetic variants linked to AUBs are also associated with a variety of plausible intermediate mechanisms. Evidence supported a strong role of drinking motives in mediating pathways between genes and broad dimensions of AUBs, with social and enhancement motives accounting for a substantial proportion of the effect of genes previously linked to higher consumption levels (DPW). Furthermore, some evidence supported the importance of specific genetic dimensions of AUBs. Genes previously associated with preference for beer and a pattern of decreasing consumption/problems (*BeerPref*) were also linked indirectly to AUBs via intermediate pathways such as low alcohol sensitivity. By simultaneously examining the role of multiple sets of genes and intermediate traits, we were able to account for a relatively high proportion of individual variation in AUBs as well as gain insight into the mechanisms by which multiple dimensions of genetic susceptibility manifest.

These results continue to support the notion that AUBs and their underlying causes are heterogeneous (Litten et al., 2015; Mallard et al., 2022; Wong & Schumann, 2008). First, genetic scores indexing multiple alcohol‐related dimensions were only moderately correlated with each other. Second, these PGSs demonstrated unique patterns of associations with intermediate measures in the univariable regression models. For example, PAU and *BeerPref* PGSs were associated with greater negative urgency, a tendency toward impulsive behavior during negative mood states, while PAU and DPW PGSs were associated with greater positive urgency, impulsive behavior during positive mood states. *BeerPref* and *AtypicalPref* also showed a unique association pattern with lower levels of conformity drinking motives and (for *BeerPref*) expectations of lower cognitive and behavioral impairment when drinking, without even marginal associations with sensation seeking, a robust risk factor for AUBs (Adams et al., 2012; Li et al., 2017; Stautz & Cooper, 2013). In combination with their association with lower alcohol sensitivity, it is plausible that the genes indexed by these factors are more specific to alcohol‐related processes. The PAU and especially DPW factors, on the other hand, show a pattern of correlations more consistent with a general externalizing/reward seeking predisposition that is robustly but nonspecifically linked to AUBs—as well as other substance use behaviors and impulsivity traits (Kendler et al., 2011; Poore et al., 2023).

Heterogeneity was also observed in the patterns of associations between alcohol‐related PGSs and measured AUBs in the mediational models. DPW PGSs were associated with all AUBs but most strongly with drinking frequency (especially in the direct genetic effects), despite drinks per week in fact being a measure of quantity moreso than frequency. Previous research has shown that genetic influences on total consumption measures such as DPW can be biased by external factors such as socioeconomic status, which have specific effects on drinking frequency rather than quantity (Mallard et al., 2022). PAU PGSs, on the other hand, were more specifically associated with AUDsx. *BeerPref* had indirect effects on Freq and AUDsx. The genetic influences on this factor might be relevant to age or developmental differences in the processes underlying AUBs since this factor represents patterns of longitudinal changes in consumption as well as alcohol problems earlier rather than later in life (i.e., individuals who may age out of drinking/drinking problems). Our results indicate that these influences are relevant to AUBs in this young adult cohort, over and above the genetic effects captured by DPW and PAU PGSs. Such dynamics are not well‐characterized in typical GWASs of static measures such as current DPW in (older) adults or a dichotomous lifetime AUD diagnosis. This factor is especially interesting because, as it indexes measures of receiving advice to reduce drinking and an actual reported decrease in drinking, it may represent biological mechanisms that facilitate recovery or are more sensitive to intervention.

Of the intermediate mechanisms investigated, the strongest evidence for mediation of genetic effects was observed for alcohol sensitivity and drinking motives. This is consistent with a large body of evidence demonstrating that these factors have robust, proximal effects on AUBs (Bresin & Mekawi, 2021; Kuntsche et al., 2005; Schuckit, 2009). However, there have been relatively few investigations of the etiology of these constructs using measured genes (Lai et al., 2020; Savage et al., 2022), and even fewer establishing whether their genetic influences overlap those of AUBs. Here, we demonstrate that many of the genetic variants linked to individual differences in AUBs appear to exert their influence via indirect pathways including alcohol sensitivity and motivations for drinking. Further, consistent with epidemiological literature (Bresin & Mekawi, 2021; Savage & Dick, 2023b), positive reinforcement social and enhancement motives mediate genetic risk for (heavy) consumption, while negative reinforcement coping motives mediate AUD risk genes, indicative of multiple distinct etiological processes. It is also interesting that the genetic influences on *BeerPref* were strongly associated with and partially mediated by alcohol sensitivity. Unlike other factors, *BeerPref* is not associated with genetic variation in the regions of the genome containing alcohol metabolism genes in the *ADH* and *ALDH* gene families (Savage et al., 2024). This suggests an important biological effect on the subjective feelings of intoxication that may be independent of direct alcohol metabolic processes, yet relevant to downstream AUBs. More biobehavioral research on the genetic etiology of these intermediate constructs is needed to enhance future therapeutic applications, for example, to reduce the rewarding effects of alcohol or dampen motivational drives for consumption.

In addition to furthering our understanding of the mechanisms underlying statistical associations between genes and AUBs, the results from this study indicate that a relatively high level of accuracy in predicting AUBs can be achieved when combining genetic indicators and intermediate traits, with models explaining 14%–36% of the between‐person variability in observed AUBs. While genetic indices such as PGSs may eventually be helpful for precision medicine, combining these with information on other risk and protective factors might also be an immediately applicable way to make interventions more effective. More specifically, while the (direct) effects of the PGSs were low, they could be used to identify individuals with higher genetic risk and target cognitive interventions toward the most relevant intermediate mechanisms, such as reshaping drinking motives and/or thoughts about the rewarding effects of alcohol. Interventions targeted toward individuals with a low alcohol sensitivity have already been shown to be useful (Savage et al., 2015; Schuckit et al., 2012), and it is even possible to use self‐reports of genetically influenced traits such as alcohol sensitivity when selecting on measured genes is not possible or not desired. Large‐scale investigations of specific AUBs are needed to continue refining their genetic etiology and lead to better personalization of such interventions. Furthermore, systematic application and evaluation are needed to determine whether personalized interventions based on genetic predispositions can improve upon the efficacy of existing programs (Strøm et al., 2014).

While promising, this study's results should be interpreted in the context of several limitations. The sample was representative of the student population from which it was derived (Dick et al., 2014), but not necessarily generalizable beyond university students or outside of the European‐like ancestry subgroup to which the sample was restricted for genetic analyses. Attrition across waves could reduce this representativeness further, although the use of measures from early waves and aggregate measures across available waves attenuates this concern somewhat. A subset of S4S participants was included in the DPW GWAS sample, making the discovery and validation cohorts not fully independent, although the overlap is minimal (S4S contributed *n* = 2314 or 0.3% of the total *N* = 666,978). Causal inferences are also not certain; although PGSs based on DNA can reasonably be assumed to come before all other measures, the direction of causation cannot be fully resolved between mediators and outcomes. Most mediators capture either stable traits (personality) or measures prior to/soon after drinking initiation (alcohol sensitivity; alcohol expectancies), but the possibility of reverse causality cannot be ruled out. It has also been demonstrated that the nature of the relationship between drinking motives and AUBs changes during the transition into young adulthood (Savage & Dick, 2023a), complicating the question of whether motives mediate the relationship between genes and AUBs or vice versa. As our tested hypotheses were entirely exploratory and limited to a single validation sample, additional replication and experimental validation are needed. Finally, while we derived PGSs from the largest available GWAS results for all measures, the effect sizes are still very low. In general, PGSs are likely to be highly underpowered in capturing genetic influences relevant to AUBs and their intermediate causes (Lewis & Vassos, 2020). It is difficult to say with certainty whether null effects of the PGSs represent a lack of association or low power due to the discovery/validation sample sizes and potential genetic heterogeneity.

In conclusion, this study highlights drinking motives, alcohol sensitivity, and, to a lesser extent, personality traits and alcohol expectancies as important mediators of the genetic influences on multiple dimensions of AUBs. Genetic indices of broad constructs such as consumption or problematic alcohol use are relevant to predicting individual‐level AUBs, as are the genetic indices of more specific AUBs involving beverage preference and consumption patterns. Additional genetic investigations of diverse AUB dimensions are needed to better understand the processes most relevant for heterogeneous groups of individuals. Further, constructs such as drinking motives and alcohol sensitivity are critical mechanisms which require further attention as endophenotypes for AUBs, especially since they are self‐report measures that are fairly easy to collect at scale. Combining genetic information with such intermediate processes has the potential to drive forward improved and personalized prevention and treatment applications.

## CONFLICT OF INTEREST STATEMENT

The authors report no competing interests.

## Data Availability

Data from this study are available to qualified researchers via dbGaP (phs001754.v4.p2) or via spit4science@vcu.edu for qualified researchers who provide the appropriate signed data use agreement.

## THE SPIT FOR SCIENCE WORKING GROUP

*Director*: Karen Chartier. *Co‐Director*: Ananda Amstadter. *Past Founding Director*: Danielle M. Dick. *Registry management*: Emily Lilley, Renolda Gelzinis, Anne Morris. *Data cleaning and management*: Katie Bountress, Amy E. Adkins, Nathaniel Thomas, Zoe Neale, Kimberly Pedersen, Thomas Bannard and Seung B. Cho. *Data collection*: Amy E. Adkins, Kimberly Pedersen, Peter Barr, Holly Byers, Erin C. Berenz, Erin Caraway, Seung B. Cho, James S. Clifford, Megan Cooke, Elizabeth Do, Alexis C. Edwards, Neeru Goyal, Laura M. Hack, Lisa J. Halberstadt, Sage Hawn, Sally Kuo, Emily Lasko, Jennifer Lend, Mackenzie Lind, Elizabeth Long, Alexandra Martelli, Jacquelyn L. Meyers, Kerry Mitchell, Ashlee Moore, Arden Moscati, Aashir Nasim, Zoe Neale, Jill Opalesky, Cassie Overstreet, A. Christian Pais, Kimberly Pedersen, Tarah Raldiris, Jessica Salvatore, Jeanne Savage, Rebecca Smith, David Sosnowski, Jinni Su, Nathaniel Thomas, Chloe Walker, Marcie Walsh, Teresa Willoughby, Madison Woodroof and Jia Yan. *Genotypic data processing and cleaning*: Cuie Sun, Brandon Wormley, Brien Riley, Fazil Aliev, Roseann Peterson and Bradley T. Webb.
